# Spatial and temporal variation in New Hampshire bat diets

**DOI:** 10.1038/s41598-022-17631-z

**Published:** 2022-08-22

**Authors:** Devon O’Rourke, Nicholas P. Rouillard, Katy L. Parise, Jeffrey T. Foster

**Affiliations:** 1 Department of Molecular, Cellular, and Biomedical Sciences, University of New Hampshire, Durham, NH USA; 2 Present Address: Pathogen and Microbiome Institute, Northern Arizona University, Flagstaff, AZ USA

**Keywords:** Behavioural ecology, Invasive species, Molecular ecology

## Abstract

Insectivorous bats consume a diverse array of arthropod prey, with diets varying by bat species, sampling location, and season. North American bat diets remain incompletely described, which is concerning at a time when many bat and insect populations appear to be declining. Understanding the variability in foraging is thus an essential component for effective bat conservation. To comprehensively evaluate local foraging, we assessed the spatial and temporal variability in prey consumed by the little brown bat, *Myotis lucifugus*, in New Hampshire, USA. We collected bat guano samples from 20 sites over 2 years and analyzed sequence data for 899 of these samples using a molecular metabarcoding approach targeting the cytochrome oxidase I subunit (COI) gene. Some prey items were broadly shared across locations and sampling dates, with the most frequently detected arthropod orders broadly similar to previous morphological and molecular analyses; at least one representative sequence variant was assigned to Coleoptera in 92% of samples, with other frequently detected orders including Diptera (73%), Lepidoptera (65%), Trichoptera (38%), and Ephemeroptera (32%). More specifically, two turf and forest pests were routinely detected: white grubs in the genus *Phyllophaga* (50%), and the Asiatic Garden beetle, *Maladera castanea* (36%). Despite the prevalence of a few taxa shared among many samples and distinct seasonal peaks in consumption of specific arthropods, diet composition varied both temporally and spatially. However, species richness did not strongly vary indicating consumption of a broad diversity of taxa throughout the summer. These data characterize little brown bats as flexible foragers adept at consuming a broad array of locally available prey resources.

## Introduction

North American insectivorous bats have highly flexible foraging strategies. Dietary analyses indicate consumption of a broad assortment of prey^1^, yet the composition of prey contents reported appears sensitive to temporal or spatial factors among little brown bats, *Myotis lucifugus*^2–4^, big brown bats, *Eptesicus fuscus*^4–6^, as well as Indiana bats, *Myotis sodalis*^7^. Similar patterns of temporal variability of arthropods in bat diets occur in their European relatives^8–10^. Thus, it is unsurprising that many of the factors associated with intraspecies variation in little brown bat diets—a species with extensive historical records—are connected with either location or season. These factors include variation in prey abundance^4,11^, bat age^12^, landscape features^13^, and ambient temperature^14^. Heterogeneity in sampling location or date can alter the community composition of prey available and therefore what is observed in bat diets^2,4,6^, suggesting that a more comprehensive understanding of the niche breadth of a species would greatly benefit from a sampling design surveying multiple populations simultaneously and repeatedly throughout the season.

The techniques used to describe diets also play a fundamental role in how we characterize the foraging habits of a species^15,16^. Historically, morphological analyses have described bat diets at the order-level due to taxonomic challenges with prey species identifications from guano^1,17^. Diet compositions of insectivorous bats in Eastern North America typically contain some ratio of Coleoptera, Diptera, and Lepidoptera, with smaller fractions of Trichoptera and Ephemeroptera and a few other taxa. For example, these visual identification methods indicate that North American bats like *E. fuscus* are beetle specialists^5^, while *Myotis* spp. more frequently consume flies and moths^18–20^. More recent studies using molecular methods support some of the historically observed order-level diet findings—*E. fuscus* still prefer beetles^6,21^ (but less so in Southwestern deserts^22^), and *M. lucifugus* continue to consume flies and moths^2,3^. Although not without their own biases (see Nielsen et al.^16^ for a review of various diet tracing techniques, and Alberdi et al.^23^ for molecular metabarcoding, specifically), molecular analyses generally have higher resolution of prey items relative to morphological techniques, and can reveal a much broader palate than previously described^24^. Rather than a small menu consisting of a few arthropod orders, molecular studies of insectivorous bats routinely describe hundreds of unique sequences detected with varying frequencies across numerous arthropod orders. In light of the superior taxonomic resolution of molecular metabarcoding, historical assessments may have significantly underestimated the niche and dietary breadth of the bat species described.

Detailed diet information provided by metabarcoding may be of particular importance for conserving North American bat populations that have exhibited drastic declines due to the fungal disease White-Nose Syndrome^25,26^. For bat populations that are recovering and for those that continue to decline, diet information can help characterize the local habitat resources required by these populations. Molecular diet information is scant for a few of the species impacted by the disease and entirely absent for others. At the same time, insect populations have recently exhibited dramatic declines worldwide^27,28^, with likely effects on aerial insectivores such as bats^9^. The first multi-year molecular analyses of the diets of North American bats observed local compositional variability within and between seasons at the same location in little brown bats^2,3^ and big brown bats^6^. Similarly, a recent evaluation of little brown bat diets across six sites in Wisconsin identified regional and local foraging preferences, with evidence of family-level diet turnover between weeks^4^. We were motivated to build on these foundational studies by significantly expanding on the number of sites sampled to better assess the potential diversity in little brown bat foraging patterns across sampling locations and years. We completed a two-year sampling regime that spanned 20 sites across southern and central New Hampshire, USA. Our study had three main objectives: first, to assess the dietary breadth of little brown bats throughout New Hampshire; second, to determine the extent with which these guano samples reveal potential forest or agricultural pests in an area; and third, to compare the extent with which bat diets vary along temporal and spatial gradients.

## Results

**Figure 1:**
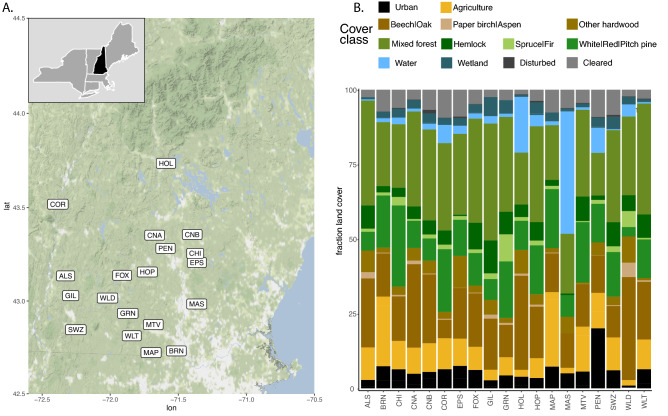
NH bat sample locations and landcover. (**a**) Guano samples collected throughout New Hampshire (dark state location of inset map) at particular locations (labels). Locations are abbreviated by 3-letter codes to reflect a particular New Hampshire town: ALS, Alsted; BRN, Brown Lane, Hollis; CHI, Chichester; CNA, Canterbury; CNB, Canterbury; COR, Cornish; EPS, Epsom; FOX, Fox State Forest, Hillsborough; GIL, Gilsum; GRN, Greenfield; HOL, Squam Science Center, Holderness; HOP, Hopkinton, MAP, Maple Hill, Hollis; MAS, Massabesic Audubon Center, Auburn; MTV, Mont Vernon; PEN, Penacook; SWZ, Swanzey; WLD, Willard Pond, Antrim; WLT, Wilton. (**b**) Fraction of land cover type within a 2500 m radius at collection site. Map created using a custom R script available at https://github.com/devonorourke/nhguano/blob/master/scripts/r_scripts/mapplots.R.

Over 4200 guano samples were collected from June to August 2015, and April to October 2016, at 20 locations throughout central and southern New Hampshire, USA. These sites included a mixture of forest land cover classes, as well as water, wetland, agricultural, and urban landscape components (Fig. 1). We generated sequence data for 2521 samples, and ultimately retained 899 samples at 19 of 20 sites for diet analyses after filtering for arthropod-specific COI sequences due to strict data quality requirements (Tables S1 and S2). Because guano samples were collected passively, classification of COI sequences was also used to identify the likely bat species. A single species, the little brown bat (*Myotis lucifugus*), was identified in 579 guano samples and contained more than 99.99% of all bat-classified sequences. Thus, while it is possible that other bat species were present and contributing guano, we did not detect them.

**Figure 2:**
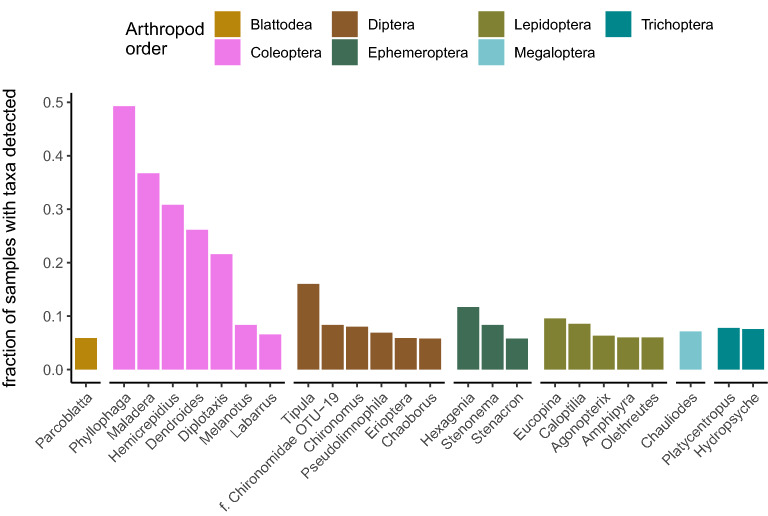
Proportion of samples with commonly detected genus labels organized by arthropod order. Genera shown represent labels present in at least 5% of samples across all New Hampshire sites. One particularly frequent taxon was missing a genus-specific label and is listed by its known family and generic OTU alias (f. Chironomidae OTU-19).

The largest fraction of arthropod sequence diversity was detected among the coleopteran order, with 766 sequence clusters (OTUs) identified among all samples, and at least one coleopteran OTU was identified among 92% of samples (Table 1). Note that ‘sequence clusters’, herein termed OTUs, are in fact denoised exact sequence variants (ASVs) clustered at 98.5% similarity (see Methods for details). Many samples also contained at least one OTU classified to Diptera (73% of samples), Lepidoptera (65%), Trichoptera (38%), and Ephemeroptera (32%). While we detected thousands of OTUs, just 51 were identified in at least 5% of samples (Table S3). Most of these frequently detected OTUs were prevalent in diets throughout most locations: 11 of the 19 sites contained at least half of these 51 OTUs. We grouped these frequent OTUs into shared genus labels rather than examining the taxonomic identity assigned to specific OTUs because several of these OTUs shared identical taxonomic labels, or alternatively, contained ambiguous species labels. Interestingly, the five most frequently detected genera are all coleopterans, and are present in at least 20% of all samples (Fig. 2). In fact, no other arthropod order had any genus represented in more than 20% of samples, although other orders contained multiple genera detected at lower frequencies. Overall, these data depict little brown bats consuming a diverse assortment of prey spanning many arthropod orders, although several beetle taxa are the most commonly consumed group in New Hampshire.

**Table 1:** Number and fraction of samples with a sequence variant (OTU).

Order	Samples detected	% Samples detected	Distinct OTUs
Coleoptera	829	0.922	766
Diptera	659	0.733	623
Lepidoptera	582	0.647	364
Trichoptera	340	0.378	111
Ephemeroptera	289	0.321	74
Hemiptera	193	0.215	102
Hymenoptera	174	0.194	99
Araneae	126	0.14	50
Megaloptera	116	0.129	38
Trombidiformes	114	0.127	57
Psocodea	80	0.089	27
Blattodea	76	0.085	9
Mesostigmata	33	0.037	13
Sarcoptiformes	31	0.034	2
Neuroptera	26	0.029	9
Odonata	14	0.016	3
Other taxa	< 10	0.026	15

OTUs are classified to a particular arthropod order among all bat guano samples collected in 2015 and 2016 throughout 19 sites in New Hampshire, USA. OTUs were clustered by collapsing exact sequence variants at 98.5% identity. Orders with fewer than ten samples detected included: Entomobryomorpha (4 samples), Plecoptera (4), Thysanoptera (3), Amphipoda (2), Dermaptera (2), Orthoptera (2), Zygentoma (2), Mantodea (1), Opiliones (1), Poduromorpha (1), and Symphypleona (1).

Two groups of beetles were frequently detected in guano samples that are listed as pests by US Forest Service (USFS) or US Department of Agriculture (USDA): white grubs of the genus *Phyllophaga* (detected in 52% of samples at 19 sites), and the Asiatic garden beetle, *Maladera castanea* (37% of samples at 18 sites). Only one other exact species match was identified as a pest in our dataset, the forest tent caterpillar moth, *Malacosoma disstria* (25 distinct samples detected at 13 sites). Because matching species identities in different databases can be complicated by ambiguous labels and incomplete records, we expanded our search to include any taxa we detected that shared the genus of a pest listed by USFS or USDA. Bats consumed a much wider variety of taxa when comparing at the genus level; 32 pest genera were identified, all of which were detected in at least 10 samples and at multiple sites (Table 2). While genus-level identification of pests is not definitive as to whether these bats are consuming particular insect species, these results suggest that bat guano sampling may provide a robust and rapid survey method for forest and agricultural pests, with refinements possible to focus on particular species.

**Table 2:** Genera detected in bat guano.

Order	Family	Genus	Samples	Sites	Detected species
Coleoptera	Scarabaeidae	**Phyllophaga**	469	19	hirsuta (399), anxia+ (216), sp. (175), longispina+ (158), hirticula+ (45), fraterna+ (36), tristis+ (29), drakii+ (12), crenulata+ (11), marginalis+ (11)
Coleoptera	Scarabaeidae	Maladera	330	18	**castanea**+ (330)
Lepidoptera	Lasiocampidae	Malacosoma	29	13	**disstria**+ (25)
Coleoptera	Elateridae	Hemicrepidius	277	18	memnonius+ (250), brevicollis* (50)
Coleoptera	Elateridae	Melanotus	204	18	hyslopi+ (107), sp. (49), similis+ (44), decumanus+ (29), communis+ (22)
Coleoptera	Scarabaeidae	Diplotaxis	199	18	sp. (199)
Coleoptera	Cerambycidae	Monochamus	51	12	notatus+ (36), sp. (14)
Coleoptera	Elateridae	Athous	49	14	brightwelli+ (37)
Coleoptera	Curculionidae	Dendroctonus	18	9	sp. (17)
Coleoptera	Curculionidae	Strophosoma	13	7	fulvicorne (13)
Diptera	Tipulidae	Tipula	245	19	sp. (203), entomophthorae (25), ultima (17), monticola (10)
Diptera	Tipulidae	Nephrotoma	28	10	sp. (11)
Hemiptera	Cicadellidae	Gyponana	33	12	sp. (28)
Hemiptera	Miridae	Blepharidopterus	16	4	provancheri+ (16)
Hemiptera	Pentatomidae	Banasa	15	7	calva+ (15)
Hemiptera	Rhyparochromidae	Ozophora	14	7	picturata+ (14)
Hymenoptera	Formicidae	Tetramorium	14	7	caespitum+ (14)
Lepidoptera	Gracillariidae	Caloptilia	94	15	alnivorella (79), sp. (13)
Lepidoptera	Tortricidae	Olethreutes	65	16	fasciatana+ (55)
Lepidoptera	Depressariidae	Psilocorsis	59	13	reflexella+ (54)
Lepidoptera	Depressariidae	Agonopterix	58	7	sp. (57)
Lepidoptera	Noctuidae	Amphipyra	54	10	pyramidoides+ (54)
Lepidoptera	Crambidae	Crambus	54	14	agitatellus (36), praefectellus (15)
Lepidoptera	Tortricidae	Epinotia	37	13	transmissana+ (14), solicitana+ (11)
Lepidoptera	Tortricidae	Pandemis	31	14	sp. (26)
Lepidoptera	Tortricidae	Argyrotaenia	30	10	quercifoliana+ (10)
Lepidoptera	Tortricidae	Cydia	30	11	latiferreana (21)
Lepidoptera	Lasiocampidae	Tolype	27	4	sp. (26)
Lepidoptera	Gelechiidae	Coleotechnites	25	9	piceaella+ (11)
Lepidoptera	Sesiidae	Synanthedon	23	10	acerni+ (23)
Lepidoptera	Blastobasidae	Hypatopa	14	6	vestaliella (11)
Lepidoptera	Tineidae	Acrolophus	13	7	sp. (13)

Pest taxa listed by the US Forest Service or US Department of Agriculture were matched at genus level to sequence variants classified in bat guano. The exact species matches are highlighted in bold. The most prevalent pest genera, *Phyllophaga*, is listed by the US Forest Service as a complex group, thus no single species is highlighted. Remaining taxa shared common genus labels only but were not exact species matches. Taxa endemic to New Hampshire are denoted (+) or endemic to other New England states as (*). Numbers of samples detected for species within shared genus listed in parentheses.

Three separate analyses investigated how bat diets varied with temporal and spatial factors: (1) comparing effect of bat diet on sampling date only, by comparing samples collected at a single site across multiple sampling windows in a single year; (2) comparing effects between multiple sites and sampling windows in a single year; (3) comparing effects of multiple sites at a single sampling date between multiple years. Sampling windows are defined in the Methods section and were used break each season into periods of equal length.

First, we compared bat diets temporally, and examined samples collected at a single site (Fox State Forest in Hillsboro, NH, USA; the site with the most complete collection data) in 2016. Sample collection spanned 6.5 months at the site, beginning in sampling window 3 on April 7 and ending in sampling window 8 on October 21 (mean 13.5 ± 9.7 S.E. samples per window). We calculated the effective number of arthropod species in samples to determine whether the dietary richness varied by sample window using three metrics (Fig. S1): observed OTUs (SR), Shannon’s entropy (H), and Faith’s phylogenetic diversity (PD). We chose to apply multiple tests to contextualize our understanding of richness: while SR provides a measure of taxa richness, H and PD provide additional information about the evenness of a community (with PD further incorporating the phylogenetic signal driving that evenness), A Kruskal–Wallis test for group differences suggested modest group differences for sampling window for all three metrics: SR (H(5) = 13.35, p = 0.02); H (H(5) = 9.79, p = 0.082); PD (H(5) = 11.99, p = 0.035). While a post hoc Dunn’s test revealed a small number of significant pairwise differences in species richness between sampling windows for uncorrected data, no significant differences were detected after applying a Benjamini–Hochberg correction. This indicates there was little variation in the richness or evenness of species detected in the sampling windows throughout the entirety of the foraging season at this site.

**Figure 3:**
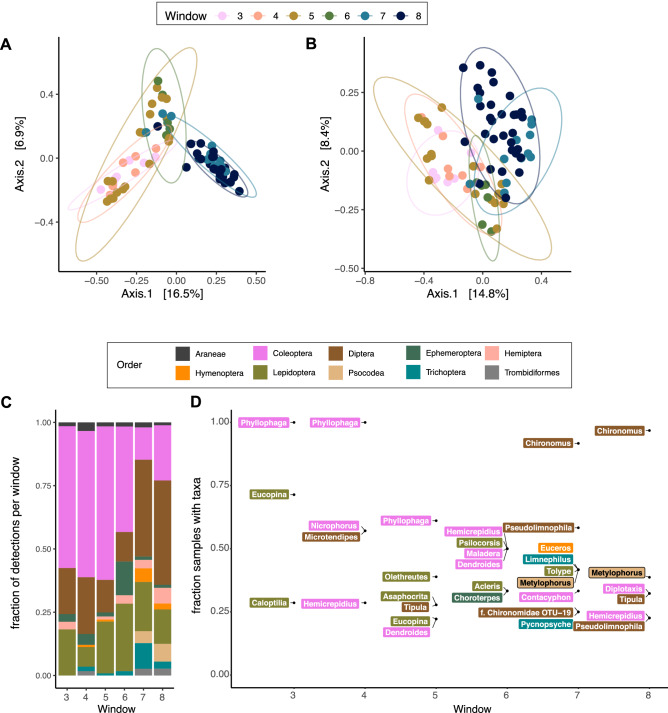
Changes in bat diet arthropod composition at one site. Compositional change at Fox State Forest (Hillsboro, NH, USA) throughout an entire foraging season in 2016. Sampling windows define 37-day periods beginning in early April and ending in late October. PCoA ordinations shown for (**a**) Dice-Sorensen index and (**b**) unweighted UniFrac distance metrics are grouped into early, mid, and late foraging season clusters; ellipses depict 95% confidence intervals around window group median. (**c**) The proportion of detections per arthropod order in a sampling window shift from being most represented by coleopteran taxa in early season to dipteran taxa in late season. (**d**) The fraction of samples with particular genera detected at each sampling window suggest specific taxa are major diet targets at different points of the foraging season. For example, coleopteran (Phyllophaga) in early sampling windows and dipteran (*Chironomus*) in late sampling windows.

However, we detected variability in diet composition, with significant main effect of sampling window for both non-phylogenetic (Dice-Sorensen; ADONIS: R^2^ = 0.22; P < 0.001; Table S4) and phylogenetic distance estimates (unweighted UniFrac; ADONIS: R^2^ = 0.19; p < 0.001) (Table S4). Evaluating diet composition using both phylogenetic along with non-phylogenetic distance measures helps contextualized what’s on the menu for these bats: with phylogenetic-weighted distances showing significant differences in diet, we understand that bats are not just eating different collections of any type of bugs, but they are consuming different collections of evolutionarily divergent types of bugs at different points of the season. Principal Coordinates Analyses (PCoA) using Dice-Sorensen (Fig. 3a) and unweighted UniFrac (Fig. 3b) distance measures clustered samples along expected temporal gradients, with early and late samples forming distinct groups between a third cluster of samples collected mid foraging season. The composition of frequently detected taxa at Fox State Forest was consistent with overall observations across New Hampshire sites, with coleopteran, dipteran, and lepidopteran orders representing the majority of OTUs detected (Fig. 3c). However, the dominant taxa shifted throughout the season, with beetle taxa being far more detected in early and mid-season samples, while flies were the most detected taxa in the later part of the foraging season. In particular, white grub beetles of the genus *Phyllophaga* were detected in the majority of samples at Fox State Forest in the early sampling windows, while non-biting midges in the genus *Chironomus* were common in later sampling windows (Fig. 3d).

We next evaluated whether sample richness and species composition varied across both location and date within one season. We focused on samples collected from seven locations in 2016 during sampling windows 4–6 (15.8 ± 9.1 samples per site + window group) due to the completeness of this dataset. Species richness varied by site + window groups for all three measures: SR (H(20) = 49.59, p < 0.001); H (H(20) = 39.06, p < 0.005); and PD (H(20) = 91.12, p < 0.001). Species richness measures were broadly similar across various site + window groups for non-phylogenetic measures, but more variable among Faith’s PD diversity (Fig. S2). A post hoc Dunn’s test for pairwise differences illustrated that only a small fraction of possible group pairs were significantly different after multiple significance correction among non-phylogenetic measures and were largely attributed to a single site + window with elevated richness (of the 210 pairwise comparisons, 7 of 10 significant differences included the 5-EPS window + site group). Similarly, significant pairwise differences in phylogenetic diversity among site + window groups were largely attributed to just 3 of 21 potential site + window groups (4MAP, 6HOL, 6PEN). In particular, 4MAP was significantly lower in diversity compared to nine other site + window groups, while 6HOL and 6PEN groups were larger in phylogenetic diversity relative to 8 and 5 site + window pairs, respectively (Figure S3). Collectively, these comparisons suggest that limited variability in dietary richness over different sampling windows and across multiple New Hampshire sites, although a few particular sites and sampling periods differed.

**Figure 4:**
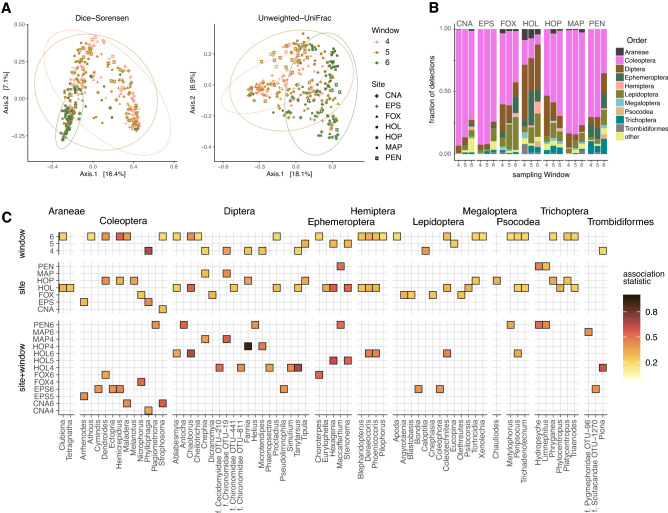
Changes in bat diet arthropod composition at multiple sites and sampling windows in 2016. (**a**) PCoA ordinations shown for Dice-Sorensen index and unweighted UniFrac distance metrics depict sampling site (point shape) or sampling window (color); ellipses depict 95% confidence intervals around window group median. (**b**) The proportion of detections per arthropod order in a sampling window for each site; arthropods with fewer than 2% of detections aggregated as “other” taxa. (**c**) Indicator species analysis performed at genus level to identify taxa associated with sampling window, or site, or site + window groups. Taxa with ambiguous genus labels identified by known arthropod family labels.

Diet composition varied in both space and time, with significant main effects for sampling widow and site, as well as their interaction, for both Dice-Sorensen and unweighted UniFrac estimates (Table S5). However, a greater proportion of variation in each PERMANOVA calculation was attributed to sampling site than sampling window for both Dice-Sorensen (Adonis: site R^2^ = 0.13, p < 0.01; window R^2^ = 0.05, p < 0.01; Table S5) and unweighted UniFrac (Adonis: site R^2^ = 0.14, p < 0.01; window R^2^ = 0.05, p < 0.01; Table S5) distances. The first two principal component axes captured only a modest proportion of variation for Dice-Sorensen (23.5%) or unweighted-UniFrac (25%) distance measures (Fig. 4a), indicating that many diet components are shared throughout these New Hampshire sites and sampling windows. Nevertheless, particular site + window groups can be discriminated by particular taxa at both the order (Fig. 4b) and genus (Fig. 4c) levels. An indicator species analysis (of shared genus labels, thus really an indicator ‘genus’ analysis) was performed separately for sampling window, site, and site + window groups (Fig. 4c) and identified representative taxa from multiple orders significantly associated with groups in each test. The majority of significant associations occur in one sampling window (window 6, 60% of significant taxa), or were associated with a single sampling site (HOL, 50% of taxa), though every distinct site or sampling window contains at least one indicator taxa, as did 13 of 21 possible site + window groups (Table S6). Notably, taxa detected in these bat guano may be associated with particular combinations of sampling window, site, or site + window groups (Figure S3), often befitting the life histories of these arthropod prey items. For example, the commonly detected genus *Phyllophaga*, fittingly known as a May or June bug, was strongly associated with sampling windows 4 and 5 (spanning April through June) but not window 6 (early July to early August).

**Figure 5:**
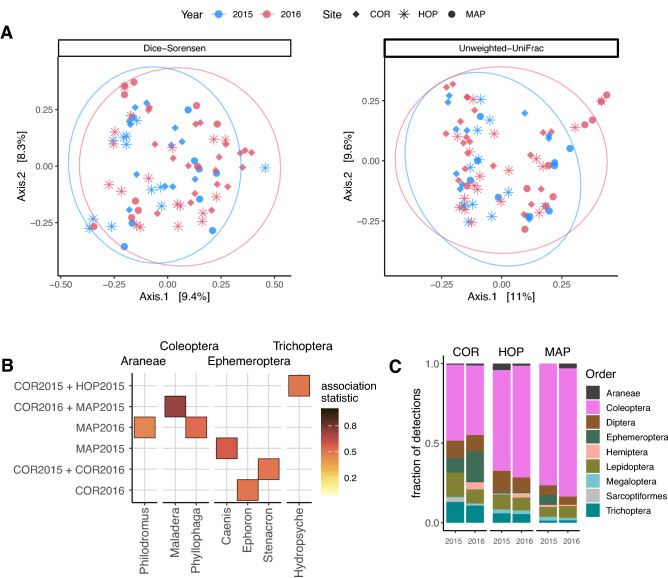
Changes in bat diet arthropod composition at multiple sites in a single sampling window between years 2015 and 2016. (**a**) PCoA ordinations shown for Dice-Sorensen index and unweighted UniFrac distance metrics depict sampling site (point shape) and sampling year (color); ellipses depict 95% confidence intervals around year group median. (**b**) Indicator species analysis performed at genus level to identify taxa associated with one or more site + year groups. (**c**) The proportion of taxa detected per arthropod order in each site + year group.

Samples collected from three sites in both 2015 and 2016 were compared within a single sampling window (window 6, July 5 to August 11) to assess the variability in bat diet richness and composition between years (13.5 ± 4.5 samples per site + year group). Species richness did not vary significantly among site + year groups for any metric: SR (H(5) = 6.54, p = 0.26); H (H(5) = 2.51, p = 0.77); and PD (H(5) = 3.56, p = 0.62). A PERMANOVA revealed that diet composition was significant for main effects of Site and Year, as well as their interaction for both metrics. However, little of the variation was captured in the model by either Dice-Sorensen (Adonis: residual R^2^ = 0.86) or Unweighted-UniFrac (Adonis: residual R^2^ = 0.87) distances. Likewise, ordinating the first two principal components of the PCoA explained a limited amount of variation for both Dice-Sorensen (17.7%) and Unweighted-UniFrac (20.6%) metrics, yet no clustering was apparent for either site, year, or site + year groups (Fig. 5a). Few taxa (at the genus level) were identified as significant indicators of a particular site + year group, with just 4 genera associated to a particular site + year, and 3 genera associated to some combination of site + year groups (Fig. 5b). Indeed, at the taxonomic order level, the proportion of taxa detected were largely consistent between years at a given site (Fig. 5c).

## Discussion

The earliest work characterizing insectivorous North American bat diets was performed using morphological techniques—even within New Hampshire specifically^19,29^—yet these visual identifications were largely limited to classifying prey to the order or family level and may have underrepresented or misassigned taxa^17^. Our study reaffirms that bats in New Hampshire are indeed foraging on many of the same arthropods described in these earlier morphological analyses: principally beetles, flies, and moths. However, our molecular approach indicates a more expansive diversity of arthropod prey than the earlier visual analyses have described, with 12 orders of insects and spiders detected in at least 5% of our samples. This was not unsurprising, as molecular metabarcoding approaches are known to reveal taxa previously unrecognized by morphological techniques^24,30^. We also detected arthropod orders like Blattodea, Psocodea, and Megaloptera, including cockroaches such as *Parcoblatta pennsylvanica*, and fishflies such as *Chauliodes pectinicornis*—taxa endemic to New Hampshire, but absent from previous bat diet records employing visual techniques. Our molecular analyses provide strong evidence that little brown bats in New Hampshire bats are capable of consuming a more diverse assortment of prey than their historical depictions.

Our most frequently detected arthropod orders (beetles, flies, and moths) were similarly depicted in previous molecular diet analyses of little brown bats^2,3^ and big brown bats^6^ from eastern Canada, as well as little brown bats in Wisconsin^4^. However, we found that the particular proportions of these arthropod components are in contrast to the preferences ascribed to these earlier reports. Coleopteran, not lepidopteran taxa, were by far the most frequently detected arthropod order in our study, despite these previous investigations indicating that beetles are preferred by big brown bats, and moths preferred by little brown bats. While it is possible that particular compositional differences occur between studies because of different primers used to generate the COI amplicon data, previous comparisons of these primer sets show no evidence for bias towards amplifying coleopteran taxa^31,32^, and would likely only reduce the breadth of taxa in earlier studies instead of the selective amplification of beetle over lepidopteran sequences. Additionally, different methodologies between studies produced similar OTU richness estimates between our single guano pellet approach, other North American insectivore richness values sampling from individual bats^22^, or earlier studies of little brown bat^3^ and big brown bat^6^ diets that used a bulk sampling (i.e., pooled guano pellets) approach. It is also possible our passive sampling technique failed to account for big brown bat contributions mixed in with little brown bat guano, as our study did not physically capture the bat to confirm species identity. However, the preponderance of bat-classified COI sequences characterizes our samples as distinctly belonging to the little brown bat, with over 99.9% of all bat-classified COI sequences assigned to the little brown bat in hundreds of guano samples. It has also been suggested that the hardy carapace of a beetle increases the likelihood of detection over softer-bodied prey items, yet the same authors also demonstrate that molecular techniques can consistently detect other arthropod orders^6^. Indeed, while we detected five distinct genera of beetles in over 20% of all samples, we also frequently detected particular genera in other arthropod orders such as Diptera. For example, dipteran sequences classified to the genus *Tipula* were detected in 144 samples, while the genus *Hexagenia* (order Ephemeroptera) were detected in 105 samples. Additionally, beetles were not universally the most dominant taxa at all sites or sampling windows. For example, samples collected in Holderness, NH contained more ephemeropteran and dipteran detections than coleopteran (see HOL in Fig. 1A). Thus, it is likely that the inordinate fondness for beetles observed among these little brown bats in New Hampshire is driven by prey availability rather than particular methodological differences.

Our extensive sampling across multiple sites and time periods was motivated to understand how spatial or temporal factors vary with New Hampshire bat diets. First, we discovered that the extent of diet composition varying with time was related to the phenology of the available prey in question. Thus, the frequency of detections for particular taxa in particular sampling windows routinely matched the expected life history of the prey. For example, beetles classified to the *Phyllophaga* genus were the most frequently detected taxa in our entire study, yet the number of samples with *Phyllophaga* detected dropped from 90% of samples between late April to late May, to 77% of samples between late May to early July, to 29% of samples between early July and mid-August. The same pattern extended to other arthropods: the psocodean barklouse (genus *Metylophorus*) was detected only in late summer and mid-fall sampling periods, befitting their seasonally late adult aerial emergence; non-biting midges like the dipteran (genus *Chironomus*) were detected in 10% or fewer samples from April through early July, yet increased from 18% of samples by mid-August, to nearly 60% of samples between August and September, an expected period of peak mating swarm activity. Collectively, our evidence supports the notion that New Hampshire bat diet composition shifts throughout the foraging season because of the variation in available prey, which is itself a function of the particular life cycle of the taxa in question. It is therefore no surprise to see May and June bugs at the top of the menu in May and June months. Systematic sampling of prey availability was beyond the scope of our study but would improve our inferences about the strength of the connections between bat diets and arthropod phenology.

The phenology of prey also explains the relatively modest effect of the sampling period on diet composition. Our samples were organized in 37-day sampling windows, a period of time shorter than the adult lifespan of many insect taxa. Thus, taxa detected across multiple sampling windows reduce the overall effect of time in our model. We observed that shifts in diet composition were the least pronounced among proximal sampling windows and the greatest during the beginning and end of the foraging season among samples collected at a single site in 2016. Likewise, when comparing across multiple sites in 2016 between three sampling periods spanning late April through mid-August, sampling period explained no more than 5% of the observed variation in diet composition.

Diet also varied based on sampling site. However, the effect of sampling location on diet composition was complicated by the fact that most sites contained highly similar land cover attributes; most sites consisted of mixed, softwood, and hardwood forest land cover. Thus, the effect of site explained no more than 14% of the observed variation in diet composition among samples collected at the seven locations evaluated in 2016. However, the greatest number of compositional differences did occur at the site with the greatest land cover difference. Samples collected from Holderness, a location less than 500 m to Squam Lake (a lake over 27 km^2^), contained more than half of all taxa uniquely associated with a particular site in an indicator species analysis, the majority of which were aquatic invertebrates. In addition, after further restricting this indicator analysis to particular site + window groups, 13 of the 23 genera significantly associated to a single group were from Holderness.

With examples of regional persistence^33,34^ providing an opportunity at conserving bat populations decimated by White-Nose Syndrome (WNS)^35,36^, diet analyses can play a consequential role informing future management considerations. The earliest U.S. Fish and Wildlife Service national plans for managing WNS specifically outlined the need to identify research methods effective for monitoring and conserving affected populations^37,38^. These kinds of guano analyses offer a rapid and robust characterization of the particular habitat resources a population requires. Our work builds on earlier studies indicating that little brown bats are sufficiently flexible in foraging across a range of land cover types^2–4^, yet it remains unclear whether this foraging flexibility is equally robust among the many other bat species affected by WNS. We recommend that future management strategies include molecular diet analyses in regional and national plans.

Beyond providing information on habitat requirements, molecular diet analyses of bat guano can also identify economic benefits bats may be providing. Bats provide well-known ecosystem services^39^, and insectivorous bats in particular can provide significant economic benefits^40^ as consumers of pests in a variety of agricultural and forested environments^31,41–43^. We discovered that two of the most ubiquitous diet components were beetle pests: the Asiatic garden beetle in 36% of samples and white pine grubs in the genus *Phyllophaga* in 48% of samples. These are turf^44^ and forest/crop^45,46^ pests, respectively, though they are of limited ecological and economic concern, and both were known to exist in New Hampshire. Among taxa detected in New Hampshire bat guano that shared a common genus with pests of concern to the USDA or USFS, all were known to exist in the Northeast, although several had not been recorded in New Hampshire specifically according to records available through the Insect and Arachnid Collections at the University of New Hampshire^47^. In some cases, it may be evident that this ambiguous classification is not a concern. For example, while there are five known species in the *Coleotechnites* genus in New Hampshire, the significant forest pest from our study, *Coleotechnites milleri,* is not one of them, and the host tree does not exist on the east coast. However, in other cases such as with the genus *Dendroctonus* it is not clear whether this ambiguous sequence is derived from one of the three endemic bark beetles, or if it represents a more concerning species such as *D. mexicanus*. Because of the ease with which guano is collected and the relatively inexpensive manner that sequence data can be obtained, molecular diet analyses can serve a dual benefit: providing a robust assessment of the dietary breadth of a species, as well as leveraging the bats’ expansive foraging capacity to screen for potential pests of concern within agency frameworks such as the USDA Forest Service early warning system^48^. While detections of putative pests discovered using this molecular approach are not definitive, and unlikely to be conclusive for species-level identification without further targeted comparisons of vouchered specimens, our results demonstrate that the broad array of arthropod orders detected using this approach can reveal pests or other non-native insects that were previously undescribed in an area.

## Methods

### Sample collection

Individual guano pellets were passively collected each week at sites throughout New Hampshire (Fig. 1) beginning June 2015 and ending October 2016. We relied on citizen scientists to assist with collecting samples at 19 of the 20 sites. Locations consisted of a mix of forest and agricultural landscapes typical of the region. We obtained data from partners collecting guano at a nature center (HOL in Fig. 1), a forest research station (FOX), conservation lands (BRN, MAP, MAS, WLD), and privately owned homes (all other sites) from bat colonies occupying structures such as attics, barns, garages, and bat houses. Volunteers were provided with supplies (forceps, dust masks, nitrile gloves, ethanol wipes, plastic sheets), and were instructed to collect up to 12 fresh guano samples per week by transferring individual pellets into the pre-filled microcentrifuge tubes containing 1 mL storage buffer (3.5 M ammonium sulfate, 16.7 mM sodium citrate, 13.3 mM EDTA, pH 5.2). Plastic sheets were replaced weekly to avoid cross contamination between weeks, and samples were shipped in batches back to our lab approximately monthly. Samples were stored at −80 °C until DNA extraction.

All research and experimental protocols were approved and performed in accordance with relevant guidelines following the University of New Hampshire’s licensing committee (IACUC) protocols 160105 and 181209. Likewise, we obtained informed consent from our citizen scientist volunteers.

### Metabarcoding

DNA was extracted from individual guano pellets using 96-well plate format of the Qiagen DNeasy PowerSoil Kit (Qiagen, Hilden, Germany) following manufacturer guidelines. Samples were eluted with 60 µL of elution buffer and up to eight extraction blanks were included per 96-well plate. Arthropod COI gene fragments were targeted for amplification using primers detailed in Jusino et al.^31^. We modified the original primer sequences to preserve the COI-specific regions, but integrated linker, pad, adapter, and barcode sequences into the oligo following Kozich et al.^49^. We used 25 µL reactions with 10 µL of extracted DNA, 1 µL each of 10 mM forward and reverse primer pairs, and 13 µL of AccuStart II PCR SuperMix (Quanta BioSciences, Gaithersburg, MD, USA). Reaction conditions consisted of an initial 2 min denaturation at 95 °C, followed by 30 cycles of 20 s at 95 °C, 15 s at 50 °C, and 60 s at 72 °C and finally a 10 min extension at 72 °C. PCR products were quantified using a PicoGreen assay (Invitrogen, Carlsbad, CA, USA) with a Tecan plate reader using excitation and emission wavelengths of 480 nm and 520 nm, respectively (Tecan Group, Männedorf, Switzerland). Samples were pooled in approximately equimolar ratios. The initial pool volume was reduced with a vacuum concentrator to approximately 2 mL and was cleaned with a QIAquick PCR purification kit (Qiagen, Hilden, Germany); libraries were eluted in 30 µL elution buffer. Libraries were quantified with a Qubit High Sensitivity assay (Thermo Fisher Scientific, Waltham, MA, USA) and fragment sizes were analyzed using TapeStation D1000 ScreenTape (Agilent Technologies, Santa Clara, CA, USA).

Libraries containing samples from 2015 were sequenced at the Hubbard Center for Genome Studies at the University of New Hampshire on an Illumina HiSeq (Illumina, San Diego, CA, USA) using v2 chemistry with 500 cycles of 2 × 250 bp paired-end reads. Samples collected in 2016 were sequenced on a MiSeq machine at TGen North using v3 chemistry with 600 cycles of 2 × 300 bp paired-end reads. Raw sequence reads are available at NCBI BioProject PRJNA560640.

### Sequence processing

Raw demultiplexed sequences were trimmed using Cutadapt v-2.3^50^ and imported into QIIME 2^51^. Sequences were denoised using DADA2 v1.10.0^52^ with the QIIME 2 q2-dada2 function ‘qiime dada2 denoise-paired’ with default settings except for the additional parameters ‘--p-trunc-len-f 181’ and ‘--p-trunc-len-r 181’, resulting in a set of representative amplicon sequence variants (ASVs) for each library. Library-specific sequences and ASV tables were merged into a single dataset using ‘qiime feature-table merge’ and ‘qiime feature-table merge-seqs’ commands, respectively. The resulting study-wide collection of ASVs were further clustered at 98.5% identity using ‘vsearch --cluster_size’ with default parameters. Note that these sequence clusters, herein referred to as operational taxonomic units (OTUs), represent the clustering of exact sequence variants, rather than a conventional notion of an OTU that is often derived from clustering raw sequence data directly.

Biological mock community samples were sequenced in eight of nine libraries shared with New Hampshire guano samples; these mock data were used in a separate experiment^32^. Both positive and negative control samples were removed from the dataset, as were the ASVs matching the expected biological mock sequences. The GitHub repository for this project provides additional details regarding data processing (‘sequence_processing.md’) and sequence quality control processes (‘contamination_evaluation.md’) https://github.com/devonorourke/nhguano/blob/master/docs/.

Representative sequences were classified using a custom database curated with reference sequences and taxonomic information obtained from the Barcode of Life Data System (BOLD) database^53^. The development and use of this curated reference set was described previously^54^. Briefly, we first obtained the BOLD COI sequences using a custom R script that queried the BOLD API using the 'bold' R package^55^. Reference sequences were filtered for nucleotide ambiguity and homopolymer runs (no more than 5 degenerate bases per sequence or 12 homopolymers) with 'qiime rescript cull-seqs', as well as for length (min 250 bp, max 1600 bp) with 'qiime rescript filter-seqs-length'. The resulting sequences and their associated taxonomic identities were dereplicated by applying a Least Common Ancestor (LCA) method that gave preference to sequences represented most frequently with 'qiime rescript dereplicate --p-mode 'super' --p-derep-prefix'. Remaining sequences were trimmed to boundaries defined by our COI primer sequences by performing multiple sequence alignment of reference and primer sequences with MAFFT^56^. The remaining primer-trimmed, nucleotide quality and length-filtered references were further filtered for length and subsequent gaps removed with 'qiime rescript degap-seqs', retaining only reference sequences with a minimum length of 170 bp. Finally, these reference sequences were dereplicated a second time with the same LCA method used earlier with 'qiime rescript dereplicate'. The database consists of 739,345 unique COI reference sequences and taxonomic labels.

We used a hybrid approach in classifying representative sequences, prioritizing exact matches from VSEARCH first, then retaining Naive Bayes classifications with sufficient information. The naive Bayes classifier was trained using the RESCRIPt command 'qiime rescript evaluate-fit-classifier', producing the QIIME object required for Naive Bayes classification. We then classified the representative sequences using VSEARCH and naive Bayes approaches separately. For VSEARCH, we required 100% identity across 94% query coverage, with 'qiime feature-classifier classify-consensus-vsearch', while default parameters were used in the naive Bayes classification process with 'qiime feature-classifier classify-sklearn'.

All bat-associated sequences classified were identified separately with naive Bayes and VSEARCH, with both read abundances and sample occurrences. The three OTUs classified as a bat by VSEARCH were identical to the taxonomic labels assigned by naive Bayes classifications, though the naive Bayes classifier had three additional distinct labels of similar bat species. While we lacked visual confirmation of species identity for all sites, these data suggest our diet analyses are restricted to the little brown bat, as it was the only bat species from this region identified in our dataset.

For both VSEARCH and naive Bayes-classified OTUs, we separately filtered the dataset to retain only those OTUs with taxonomic family information assigned to phylum "Arthropoda". Thus, an OTU may be included that lacked genus or species labels, provided it retained an unambiguous family label. To select a final list of OTUs, we first retained VSEARCH-classified OTUs that fit these filtering criteria. Among VSEARCH-classified OTUs that did not pass this filtering threshold, we then selected from the naive Bayes-classified OTUs that met the same standard, when possible. In all, 559 OTUs were selected using the VSEARCH method initially, and 2,627 subsequently from the naive Bayes method. We manually resolved a single label that was undefined by either classification method, OTU-1, which was classified as *M. castanea* after using NCBI BLAST^57^ and discovering an exact match. Among all OTUs remaining across all samples, we next normalized samples using a method of scaling with ranked subsampling using the SRS R package^58^. We retained only those samples (and their OTUs) with a per-sample minimum of 1000 arthropod-classified reads for subsequent diversity analyses. These sequences were further used to generate a rooted tree using the function 'qiime phylogeny align-to-tree-mafft-fasttree', applied for all phylogenetic-based estimates (Faith’s phylogenetic diversity and unweighted UniFrac).

### Diversity analyses

Diversity analyses were conducted among all guano samples to identify the most frequent taxa consumed by little brown bats throughout New Hampshire, as well as among particular subsets of samples to evaluate whether dietary richness and community composition varied among with particular collection sites or dates. Samples were grouped into 37-day sampling windows to maximize the greatest number of samples in the smallest range of time shared across the most locations that generated sufficient sequence data (Table S2). For example, window 3 was a sampling period from March 16 to April 22, window 4 was April 23 to May 29, and window 8 was September 17 through October 24. Windows with a shorter time period, say monthly, did not contain enough samples to robustly compare across the season or spatially. We applied the same methods for each spatial and/or temporal investigation. Samples were assessed for species richness using three metrics: observed OTUs, Shannon’s entropy, and Faith’s phylogenetic diversity. Kruskal–Wallis tests were then applied to evaluate group differences, and a post hoc Dunn’s test (with Benjamini–Hochberg correction) assessed the statistical significance of particular pairwise comparisons between groups. Community composition was calculated following a binary presence-absence transformation of sequence counts with Dice-Sorensen and Unweighted UniFrac distance measures. We evaluated differences in group medians and group dispersions with ‘adonis2’ and ‘betadisper’ functions in Vegan^59^, respectively. Principal coordinates analyses (PCoA) were completed with the ‘ordinate’ function in Phyloseq^60^ for each distance matrix. Indicator species analyses were completed with the R function ‘multipatt’ from the R ‘indicspecies’ package^61^, and restricted our analyses to those arthropod orders detected in at least 2% of samples in at least one group. Further descriptions of the bioinformatic steps, associated scripts, and related files for database construction, classification, and diversity analyses are available in the ‘diversity_analyses’.md’ document in the project GitHub repository (see Data Accessibility below).

### Pest analysis

To identify whether taxa classified in our dataset were considered forest or agricultural pests, we cross referenced lists maintained by the U.S. Forest Service (USFS) and the U.S. Department of Agriculture (USDA). We used a custom R script, pest_work.R, to perform the comparisons, which were restricted to first identifying how many sequence variants were exact species matches, then expanding the search to identify instances in which common genera were shared. This was done because there were instances in which the USDA did listed taxa as ambiguous species (e.g., *Malacosoma* sp*.*) thus even if we classified our taxa to a species level, an exact match was not possible.

### Additional software

Note that additional software within the QIIME environment such as Pandas^62^ and BIOM^63^ were used, as was a suite of R libraries including ape^64^, btools^65^, cowplot^66^, decontam^67^, FSA^68^, qiime2R^69^, ggmap^70^, ggpubr^71^, ggrepel^72^, indicspecies^61^, landscapemetrics^73^, lubridate^74^, magicfor^75^, Matrix^76^, multcompView^77^, pairwiseAdonis^78^, phyloseq^60^, raster^79^, reshape2^80^, scales^81^, sf^82^, SRS^58^, svglite^83^, tidyverse^84^, urbnmapr^85^, usedist^86^, and vegan^59^.

## Supplementary Information

Supplementary Information.

## Data Availability

Data are provided as private-for-peer review (shared publicly). All scripts, supplementary figures and tables, and metadata is currently stored at the following GitHub repository: https://github.com/devonorourke/nhguano**.** Large individual database files used for classification are available in the following directory hosted by Open Science Framework: https://osf.io/d4jra/. Raw sequence reads are published and currently hosted at NCBI BioProject PRJNA560640. The GitHub repository is the intended location where data will be permanently archived if the paper is accepted for publication. This submission uses novel code, which is provided in an external repository to be evaluated during the peer review process. Please see the scripts directory of the project’s GitHub repository: https://github.com/devonorourke/nhguano/tree/master/scripts. Supplementary tables and figures referred to within the manuscript are available at: https://github.com/devonorourke/nhguano/tree/master/supplementaryData.
