# Supplementary material for: Spatial and temporal variation in New Hampshire bat diets
Source: Sci Rep. 2022 Aug 22;12:14334. doi: 10.1038/s41598-022-17631-z (PMC9395357; doi:10.1038/s41598-022-17631-z)
Supplement: Supplementary file 1 — Supplementary Information. [file 41598_2022_17631_MOESM1_ESM.docx]

**Supplemental Information for:**

Spatial and temporal variation in New Hampshire bat diets

Devon O’Rourke, Nicholas P. Rouillard, Katy L. Parise, Jeffrey T. Foster

**Table of Contents:**

| **Figure S1** | Page 2 |
| --- | --- |
| **Figure S2** | Page 3 |
| **Figure S3** | Page 4 |
| **Table S1** | Page 5 |
| **Table S2** | Page 6 |
| **Table S3** | Page 7-8 |
| **Table S4** | Page 9 |
| **Table S5** | Page 9 |
| **Table S6** | Page 11 |

**Figure S1.** Dietary richness for a single site (Fox State Forest in Hillsboro, NH, USA) in 2016 among samples collected in six time periods: sampling windows span 37 day periods, beginning in Window 3 (March 16 to April 22) and ending in Window 8 (September 17 through October 24). Richness shown for three metrics: observed OTUs (SR), Shannon’s entropy (H), and Faith’s phylogenetic diversity (PD).


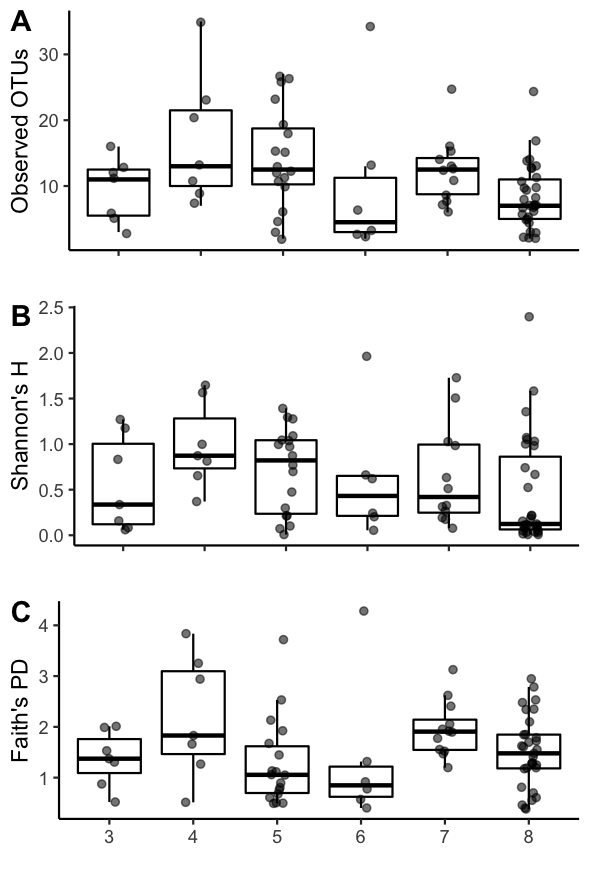


**Figure S2.** Dietary richness among samples collected at one of six sites in 2016 during three sampling windows beginning on Window 4 (April 23 to May 29) and ending on Window 6 (July 5 to August 11). Richness shown for three metrics: observed OTUs (SR), Shannon’s entropy (H), and Faith’s phylogenetic diversity (PD). Pairwise significant differences between each site+date group indicated by distinct letters above each group.


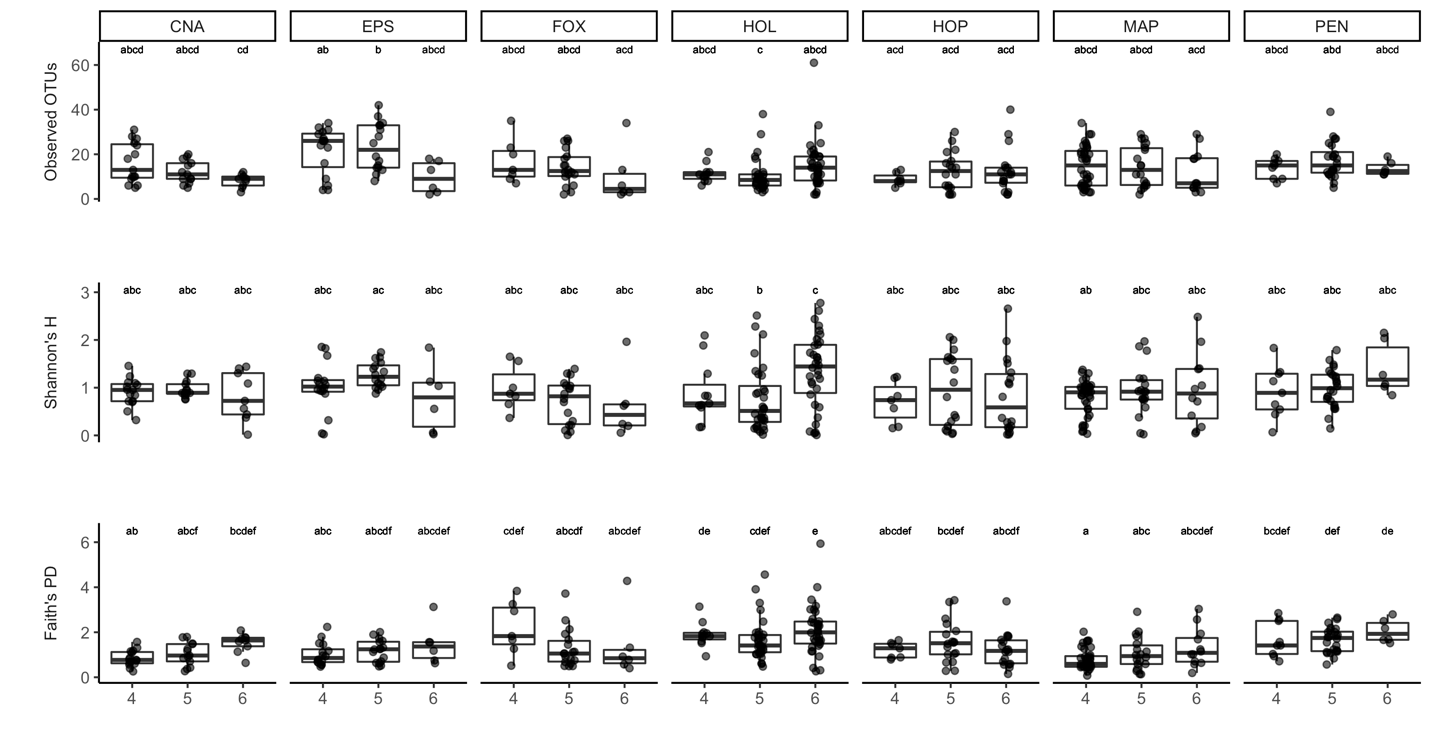


**Figure S3.** Indicator species analysis among samples collected at one of six sites in 2016 during three sampling windows beginning on Window 4 (April 23 to May 29) and ending on Window 6 (July 5 to August 11). Analysis to identify particular diet items distinguishing a group conducted separately for sites only, sampling windows only, and for site+window groups.


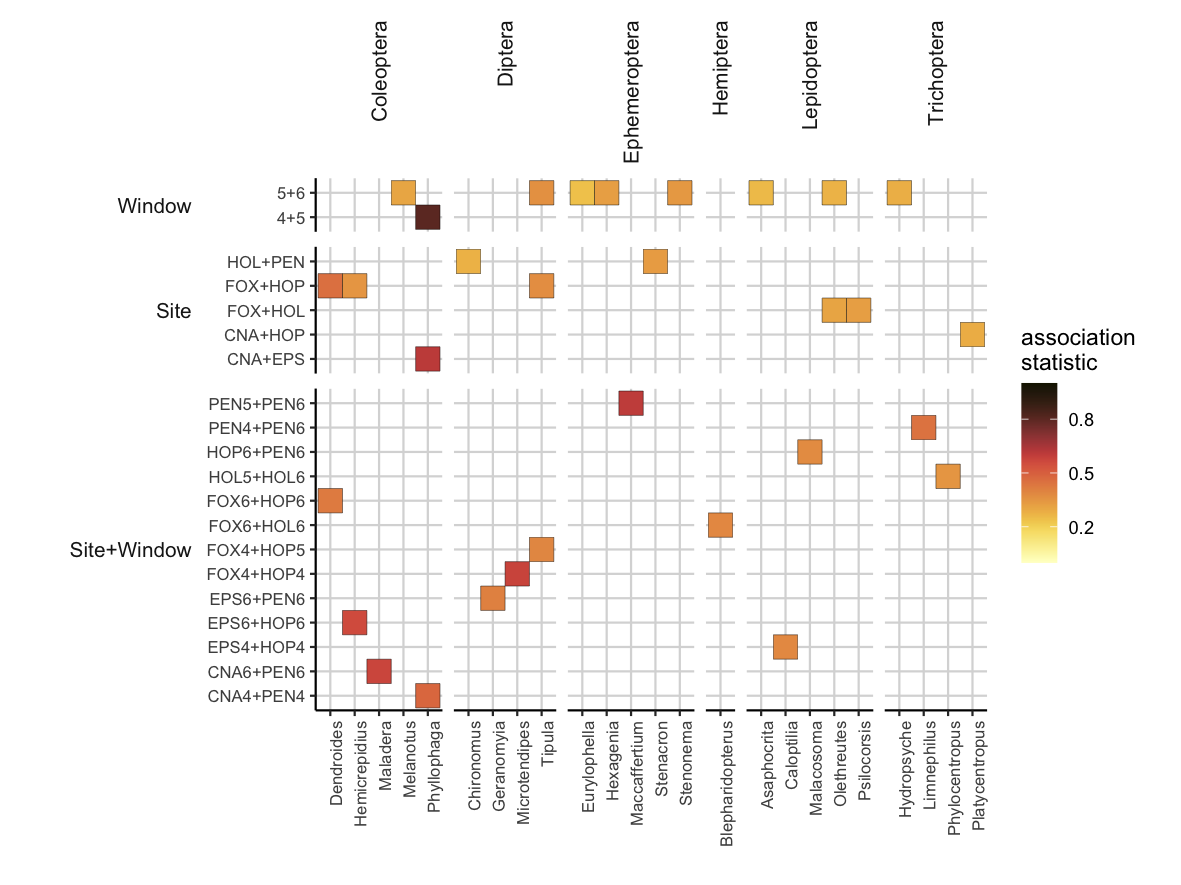


**Table S1.** Comparison the number of samples collected relative to number of samples that passed quality controls for analyses in study, per site, per year species.

| Site | collected2015 | evaluated2015 | collected2016 | evaluated2016 |
| --- | --- | --- | --- | --- |
| ALS | .. | .. | 150 | 43 |
| BRN | 144 | 11 | 264 | 49 |
| CHI | .. | .. | 240 | 46 |
| CNA | .. | .. | 122 | 38 |
| CNB | .. | .. | 194 | 38 |
| COR | 71 | 20 | 73 | 24 |
| EPS | .. | .. | 240 | 42 |
| FOX | 94 | 16 | 337 | 82 |
| GIL | 23 | 5 | 48 | 16 |
| GRN | 48 | 2 | .. | .. |
| HOL | .. | .. | 264 | 80 |
| HOP | 191 | 28 | 335 | 58 |
| MAP | 140 | 16 | 252 | 73 |
| MAS | 82 | 12 | 188 | 4 |
| MTV | .. | .. | 203 | 68 |
| PEN | .. | .. | 118 | 39 |
| ROL | .. | .. | 24 | .. |
| SWZ | 139 | 45 | .. | .. |
| WLD | 131 | 23 | .. | .. |
| WLT | 105 | 21 | .. | .. |

**Table S2.** Comparison of number of samples available for analysis per site per year per sampling window. Top row represents sampling year (2015 and 2016), second row represents sampling window (5-8, and 3-9). Sampling windows span 37 day periods, beginning in Window 3 (March 16 to April 22) and ending in Window 8 (September 17 through October 24).

|  | 2015 | | | | 2016 | | | | | | |
| --- | --- | --- | --- | --- | --- | --- | --- | --- | --- | --- | --- |
| Site | **5** | **6** | **7** | **8** | **3** | **4** | **5** | **6** | 7 | 8 | 9 |
| ALS | 0 | 0 | 0 | 0 | 0 | 0 | 0 | 18 | 22 | 3 | 0 |
| BRN | 0 | 5 | 6 | 0 | 0 | 30 | 14 | 4 | 1 | 0 | 0 |
| CHI | 0 | 0 | 0 | 0 | 0 | 0 | 12 | 15 | 19 | 0 | 0 |
| CNA | 0 | 0 | 0 | 0 | 0 | 15 | 13 | 10 | 0 | 0 | 0 |
| CNB | 0 | 0 | 0 | 0 | 0 | 4 | 17 | 8 | 9 | 0 | 0 |
| COR | 2 | 12 | 6 | 0 | 0 | 0 | 0 | 20 | 4 | 0 | 0 |
| EPS | 0 | 0 | 0 | 0 | 0 | 16 | 16 | 6 | 4 | 0 | 0 |
| FOX | 3 | 8 | 5 | 0 | 7 | 7 | 18 | 6 | 12 | 31 | 1 |
| GIL | 0 | 3 | 0 | 2 | 0 | 10 | 6 | 0 | 0 | 0 | 0 |
| GRN | 2 | 0 | 0 | 0 | 0 | 0 | 0 | 0 | 0 | 0 | 0 |
| HOL | 0 | 0 | 0 | 0 | 0 | 11 | 32 | 34 | 2 | 1 | 0 |
| HOP | 5 | 13 | 6 | 4 | 5 | 7 | 18 | 18 | 0 | 10 | 0 |
| MAP | 3 | 9 | 4 | 0 | 0 | 36 | 18 | 12 | 0 | 7 | 0 |
| MAS | 1 | 10 | 1 | 0 | 0 | 1 | 1 | 2 | 0 | 0 | 0 |
| MTV | 0 | 0 | 0 | 0 | 0 | 0 | 22 | 13 | 16 | 17 | 0 |
| PEN | 0 | 0 | 0 | 0 | 0 | 9 | 24 | 6 | 0 | 0 | 0 |
| SWZ | 5 | 24 | 16 | 0 | 0 | 0 | 0 | 0 | 0 | 0 | 0 |
| WLD | 6 | 15 | 2 | 0 | 0 | 0 | 0 | 0 | 0 | 0 | 0 |
| WLT | 5 | 4 | 12 | 0 | 0 | 0 | 0 | 0 | 0 | 0 | 0 |

**Table S3.** Table of most frequently detected OTUs (exact sequence variants clustered at 98.5% identity). Showing the number and fraction of OTUs detected across all samples in the study along with taxonomic information (taxonomic Order, Genus, and Species, when applicable), as well as the OTU alias used as the identifier through the manuscript. OTUid represents the QIIME-specific alphanumeric key assigned to a unique OTU, which can be used when accessing raw data records available in the Zenodo archive.

| **Order** | **Genus** | **Species** | **# Samples with OTU** | **% Samples with OTU** | **OTUalias** | **OTUid** |
| --- | --- | --- | --- | --- | --- | --- |
| Blattodea | Parcoblatta | Parcoblatta uhleriana | 53 | 0.05895439 | OTU-49 | 7cee309d88263cb90811fe24f627aeae |
| Coleoptera | Dendroides | Dendroides canadensis | 181 | 0.20133482 | OTU-10 | 92e807f319442dd87ddae1a3f7725e65 |
| Coleoptera | Dendroides | Dendroides canadensis | 47 | 0.05228031 | OTU-153 | 15026701e67c14acc6da34251e1b8ec4 |
| Coleoptera | Dendroides | Dendroides canadensis | 45 | 0.05005562 | OTU-168 | 0bb64283957e498be2b9cd8fa3d18c95 |
| Coleoptera | Dendroides | Dendroides concolor | 60 | 0.06674082 | OTU-50 | 04f0c5769fcb09d7d91f7e73a349518c |
| Coleoptera | Diplotaxis | NA | 183 | 0.20355951 | OTU-9 | dacf098cf73e7cf3e16a1d89287188f6 |
| Coleoptera | Diplotaxis | NA | 59 | 0.06562848 | OTU-57 | 873a3d186ca2bfa1fc2c503b59c0d0e9 |
| Coleoptera | Diplotaxis | NA | 58 | 0.06451613 | OTU-103 | a7b90c8d4da7f7087ba278f514194ca6 |
| Coleoptera | Hemicrepidius | Hemicrepidius brevicollis | 50 | 0.05561735 | OTU-54 | 44ff1f1258c5ae73633dd2536fc3a50b |
| Coleoptera | Hemicrepidius | Hemicrepidius memnonius | 250 | 0.27808676 | OTU-4 | 80c4d80457fcc8395bbccc4f364533a8 |
| Coleoptera | Labarrus | Labarrus cincticulus | 59 | 0.06562848 | OTU-62 | c4afba60302c265bd32b9ef5846b7377 |
| Coleoptera | Maladera | Maladera castanea | 330 | 0.36707453 | OTU-1 | c5098366c5ba2170bb0bdbb4ebb969cf |
| Coleoptera | Melanotus | Melanotus hyslopi | 75 | 0.08342603 | OTU-132 | ddfa0472353451578d9575aab094716a |
| Coleoptera | Phyllophaga | Phyllophaga anxia | 203 | 0.22580645 | OTU-7 | dd8ead02d602f21e3650a1dfafdbf815 |
| Coleoptera | Phyllophaga | Phyllophaga anxia | 52 | 0.05784205 | OTU-73 | 6eaae078420e552cc363c5f4780aa6fc |
| Coleoptera | Phyllophaga | Phyllophaga hirsuta | 378 | 0.42046719 | OTU-2 | 8aa059190885cb9440084a9821d998bd |
| Coleoptera | Phyllophaga | Phyllophaga hirsuta | 244 | 0.27141268 | OTU-6 | 9b325409d4145b3b0e225a2898c95d4e |
| Coleoptera | Phyllophaga | Phyllophaga hirsuta | 146 | 0.16240267 | OTU-45 | dfca7860c63a33d7c089f71282707255 |
| Coleoptera | Phyllophaga | Phyllophaga hirsuta | 116 | 0.12903226 | OTU-110 | ec9f8a7eb5d4108a2dbf70de19089914 |
| Coleoptera | Phyllophaga | Phyllophaga hirsuta | 112 | 0.12458287 | OTU-98 | 581908a907d4a43569ff7ab27e81d92f |
| Coleoptera | Phyllophaga | Phyllophaga hirsuta | 80 | 0.08898776 | OTU-141 | 4520bec9094a2cd39253c1583cd34316 |
| Coleoptera | Phyllophaga | Phyllophaga hirsuta | 79 | 0.08787542 | OTU-56 | 164c6d1dc6dc62f3a5a9ab952da44f11 |
| Coleoptera | Phyllophaga | Phyllophaga hirsuta | 69 | 0.07675195 | OTU-155 | cfca0b93157741b1761a48c2ef432007 |
| Coleoptera | Phyllophaga | Phyllophaga hirsuta | 56 | 0.06229143 | OTU-147 | 4995a74b93fbcbd4863b8c90d9a203e3 |
| Coleoptera | Phyllophaga | Phyllophaga hirsuta | 49 | 0.05450501 | OTU-247 | ee3a6834c427e1b13790f3cffa05b7e5 |
| Coleoptera | Phyllophaga | Phyllophaga hirsuta | 47 | 0.05228031 | OTU-184 | 69b5f37bc81d188104e8bb4752dcf91a |
| Coleoptera | Phyllophaga | Phyllophaga longispina | 111 | 0.12347052 | OTU-84 | 3449f6fcaac748b5a36c5669bd41c07c |
| Coleoptera | Phyllophaga | Phyllophaga longispina | 95 | 0.10567297 | OTU-89 | aafdb90962b5cf3f0dec2723b4a51d1e |
| Coleoptera | Phyllophaga | NA | 121 | 0.13459399 | OTU-93 | 7a7a22fee1f403b540ef56bdd72730a3 |
| Coleoptera | Phyllophaga | NA | 121 | 0.13459399 | OTU-95 | c805d4e616ccda7232cf489692e7448f |
| Coleoptera | Phyllophaga | NA | 98 | 0.10901001 | OTU-129 | 31a28ac06c612e1cffa640ba7458a875 |
| Coleoptera | Phyllophaga | NA | 92 | 0.10233593 | OTU-122 | 129778c8e844bed04c1422b5a25ad7a5 |
| Coleoptera | Phyllophaga | NA | 46 | 0.05116796 | OTU-257 | 726042bb37839f5b9b9337e9ba2d2606 |
| Diptera | Chaoborus | Chaoborus punctipennis | 52 | 0.05784205 | OTU-53 | af2ab83b85a6265e8a1e56410e308ebf |
| Diptera | Chironomus | NA | 72 | 0.08008899 | OTU-15 | d60fd5104a3b25d195a9f6b2f97879b8 |
| Diptera | Erioptera | Erioptera caliptera | 53 | 0.05895439 | OTU-12 | d18b9b0d9c0501fc4310fd74dbfc5a16 |
| Diptera | Pseudolimnophila | NA | 62 | 0.06896552 | OTU-17 | ac6327a1ae9883cd711b1540091ab15e |
| Diptera | Tipula | NA | 78 | 0.08676307 | OTU-36 | 2fcdfe59f27b507c180020747d4c77da |
| Diptera | Tipula | NA | 74 | 0.08231368 | OTU-68 | 61d776536df85afa044e4b7b6f7ea839 |
| Diptera | NA | NA | 75 | 0.08342603 | OTU-19 | 1b18a4671325538a4ab7b6f3f4315195 |
| Ephemeroptera | Hexagenia | Hexagenia limbata | 105 | 0.11679644 | OTU-16 | 3bccc7419ed71054e8d594ffa35fe0c6 |
| Ephemeroptera | Stenacron | Stenacron interpunctatum | 52 | 0.05784205 | OTU-61 | 2050295dd1918980f7eb677368198625 |
| Ephemeroptera | Stenonema | Stenonema femoratum | 75 | 0.08342603 | OTU-14 | d52aa99985f4de1593549d5f2e0b7cbd |
| Lepidoptera | Agonopterix | NA | 57 | 0.06340378 | OTU-3 | 73d24c9b0eca55bcce443e60c5639b37 |
| Lepidoptera | Amphipyra | Amphipyra pyramidoides | 54 | 0.06006674 | OTU-29 | eb3a4a579b410351b538a2510c37a56c |
| Lepidoptera | Caloptilia | Caloptilia alnivorella | 77 | 0.08565072 | OTU-18 | 2224f202213b63a097f0a8b32859850f |
| Lepidoptera | Eucopina | Eucopina tocullionana | 86 | 0.09566185 | OTU-13 | bb7a70bd300d349afb79afa8797e76e7 |
| Lepidoptera | Olethreutes | Olethreutes fasciatana | 54 | 0.06006674 | OTU-38 | 1599b1cb541938729b721f96e6118499 |
| Megaloptera | Chauliodes | Chauliodes pectinicornis | 64 | 0.07119021 | OTU-127 | df673a0b19267b9b666b868a983f14fa |
| Trichoptera | Hydropsyche | Hydropsyche sparna | 68 | 0.0756396 | OTU-71 | e1220ee8629ff7ecaff92f6010a848d9 |
| Trichoptera | Platycentropus | Platycentropus radiatus | 70 | 0.07786429 | OTU-25 | 65c16f3bd80e055bfeff00f11a6fade1 |

**Table S4.** PERMANOVA analysis investigating main effects of sampling period (Window) at a single site: Fox State Forest in Hillsboro, NH, USA; the site with the most complete collection data, for 2016 only. Showing PERMANOVA results for Adonis test using a distance metric without phylogenetic information (Dice-Sorensen) and with phylogenetic signal (UniFrac Unweighted).

| Class | Df | Sum.Sq | F.value | R2 | Pr..F. | Metric |
| --- | --- | --- | --- | --- | --- | --- |
| Window | 5 | 7.709 | 4.311 | 0.223 | 0.001 | Dice-Sorensen |
| Residual | 75 | 26.823 | NA | 0.777 | NA | Dice-Sorensen |
| Total | 80 | 34.532 | NA | 1 | NA | Dice-Sorensen |
| Window | 5 | 4.686 | 3.429 | 0.186 | 0.001 | UniFrac Unweighted |
| Residual | 75 | 20.503 | NA | 0.814 | NA | UniFrac Unweighted |
| Total | 80 | 25.189 | NA | 1 | NA | UniFrac Unweighted |

**Table S5.** PERMANOVA analysis investigating main effects of diet composition across multiple sampling sites (Site) and sampling periods (Window) for 2016 only. Showing PERMANOVA results for Adonis test using a distance metric without phylogenetic information (Dice-Sorensen) and with phylogenetic signal (UniFrac Unweighted).

| Class | Df | Sum.Sq | R2 | F.value | Pr..F. | Metric | WindowGroup |
| --- | --- | --- | --- | --- | --- | --- | --- |
| Site | 6 | 17.231 | 0.126 | 8.626 | 0.001 | Dice-Sorensen | windows456 |
| Window | 2 | 6.533 | 0.048 | 9.81 | 0.001 | Dice-Sorensen | windows456 |
| Site:Window | 12 | 10.031 | 0.073 | 2.511 | 0.001 | Dice-Sorensen | windows456 |
| Residual | 310 | 103.214 | 0.753 | NA | NA | Dice-Sorensen | windows456 |
| Total | 330 | 137.009 | 1 | NA | NA | Dice-Sorensen | windows456 |
| Site | 6 | 13.7 | 0.139 | 9.689 | 0.001 | UniFrac-Unweighted | windows456 |
| Window | 2 | 4.946 | 0.05 | 10.495 | 0.001 | UniFrac-Unweighted | windows456 |
| Site:Window | 12 | 7.064 | 0.072 | 2.498 | 0.001 | UniFrac-Unweighted | windows456 |
| Residual | 310 | 73.056 | 0.74 | NA | NA | UniFrac-Unweighted | windows456 |
| Total | 330 | 98.766 | 1 | NA | NA | UniFrac-Unweighted | windows456 |

**Table S6.** Indicator species analysis showing the number of genus-level taxa significantly associated to a particular Site and/or Sampling Window. For example, the site at Holderness, NH (HOL) contained the largest number of genera associated to that location (18) at any sampling date, whereas sampling window 6 contained the largest number of genera associated at a particular time interval across any sampling location.

| Group | Index | # Significant Taxa |
| --- | --- | --- |
| Site | HOL | 18 |
| Site | FOX | 5 |
| Site | HOP | 5 |
| Site | FOX+HOP | 3 |
| Site | PEN | 3 |
| Site | FOX+HOL | 2 |
| Site | HOL+PEN | 2 |
| Site | MAP | 2 |
| Site | CNA | 1 |
| Site | CNA+EPS | 1 |
| Site | CNA+HOP | 1 |
| Site | EPS | 1 |
| Site+Window | HOL4 | 6 |
| Site+Window | HOL6 | 5 |
| Site+Window | HOL5 | 2 |
| Site+Window | PEN6 | 2 |
| Site+Window | CNA4+PEN4 | 1 |
| Site+Window | CNA6 | 1 |
| Site+Window | CNA6+PEN6 | 1 |
| Site+Window | EPS4+HOP4 | 1 |
| Site+Window | EPS5 | 1 |
| Site+Window | EPS6 | 1 |
| Site+Window | EPS6+HOP6 | 1 |
| Site+Window | EPS6+PEN6 | 1 |
| Site+Window | FOX4 | 1 |
| Site+Window | FOX4+HOP4 | 1 |
| Site+Window | FOX4+HOP5 | 1 |
| Site+Window | FOX6 | 1 |
| Site+Window | FOX6+HOL6 | 1 |
| Site+Window | FOX6+HOP6 | 1 |
| Site+Window | HOL5+HOL6 | 1 |
| Site+Window | HOP4 | 1 |
| Site+Window | HOP6+PEN6 | 1 |
| Site+Window | MAP4 | 1 |
| Site+Window | MAP6 | 1 |
| Site+Window | PEN4+PEN6 | 1 |
| Site+Window | PEN5+PEN6 | 1 |
| Window | 6 | 26 |
| Window | 5+6 | 8 |
| Window | 4 | 7 |
| Window | 4+5 | 1 |
| Window | 5 | 1 |
